# SARS-CoV-2 Vaccination and the Multi-Hit Hypothesis of Oncogenesis

**DOI:** 10.7759/cureus.50703

**Published:** 2023-12-17

**Authors:** Raquel Valdes Angues, Yolanda Perea Bustos

**Affiliations:** 1 Neurology, Oregon Health and Science University School of Medicine, Portland, USA; 2 Education, Generalitat de Catalunya, Barcelona, ESP

**Keywords:** oncogenesis, metastasis, recurrence, malignancy, vaccines, spike glycoprotein, sars-cov-2, covid-19, cancer

## Abstract

Cancer is a complex and dynamic disease. The “hallmarks of cancer” were proposed by Hanahan and Weinberg (2000) as a group of biological competencies that human cells attain as they progress from normalcy to neoplastic transformation. These competencies include self-sufficiency in proliferative signaling, insensitivity to growth-suppressive signals and immune surveillance, the ability to evade cell death, enabling replicative immortality, reprogramming energy metabolism, inducing angiogenesis, and activating tissue invasion and metastasis. Underlying these competencies are genome instability, which expedites their acquisition, and inflammation, which fosters their function(s). Additionally, cancer exhibits another dimension of complexity: a heterogeneous repertoire of infiltrating and resident host cells, secreted factors, and extracellular matrix, known as the tumor microenvironment, that through a dynamic and reciprocal relationship with cancer cells supports immortality, local invasion, and metastatic dissemination. This staggering intricacy calls for caution when advising all people with cancer (or a previous history of cancer) to receive the COVID-19 primary vaccine series plus additional booster doses. Moreover, because these patients were not included in the pivotal clinical trials, considerable uncertainty remains regarding vaccine efficacy, safety, and the risk of interactions with anticancer therapies, which could reduce the value and innocuity of either medical treatment.

After reviewing the available literature, we are particularly concerned that certain COVID-19 vaccines may generate a pro-tumorigenic milieu (i.e., a specific environment that could lead to neoplastic transformation) that predisposes some (stable) oncologic patients and survivors to cancer progression, recurrence, and/or metastasis. This hypothesis is based on biological plausibility and fulfillment of the multi-hit hypothesis of oncogenesis (i.e., induction of lymphopenia and inflammation, downregulation of angiotensin-converting enzyme 2 (ACE2) expression, activation of oncogenic cascades, sequestration of tumor suppressor proteins, dysregulation of the RNA-G quadruplex-protein binding system, alteration of type I interferon responses, unsilencing of retrotransposable elements, etc.) together with growing evidence and safety reports filed to Vaccine Adverse Effects Report System (VAERS) suggesting that some cancer patients experienced disease exacerbation or recurrence following COVID-19 vaccination. In light of the above and because some of these concerns (i.e., alteration of oncogenic pathways, promotion of inflammatory cascades, and dysregulation of the renin-angiotensin system) also apply to cancer patients infected with SARS-CoV-2, we encourage the scientific and medical community to urgently evaluate the impact of both COVID-19 and COVID-19 vaccination on cancer biology and tumor registries, adjusting public health recommendations accordingly.

## Introduction and background

A number of estimates and modeling studies highlight the millions of lives that COVID-19 vaccines might have saved globally [1-6]. Yet, the COVID-19 crisis has negatively impacted the health and well-being of many people, particularly those living with cancer (i.e., the COVID-19 pandemic disrupted access to medical care, postponed cancer screenings and diagnostic and therapeutic services, separated cancer patients from family and loved ones, and became an additional source of stress and anguish). Three years into the pandemic, healthcare authorities keep recommending that people with active and prior cancer get vaccinated against COVID-19 [7]. Booster doses are encouraged [7,8] because vaccine effectiveness wanes with time [9] and some cancers and cancer treatments affect the immune system, rendering the vaccines less efficient [10]. While clinical trials for COVID-19 vaccines overlooked patients with cancer [11-15], the assumption is that those with a compromised immune system are at higher risk for severe disease, so getting even some protection from the vaccine is better than no protection. However, a growing body of evidence [16-21] suggests that some individuals with active or prior cancer experienced disease exacerbation following COVID-19 vaccination. Reports registered in the Vaccine Adverse Effects Report System (VAERS) [22], a national self-reporting vaccine safety surveillance system comanaged by the US Centers for Disease Control and Prevention (CDC) and US Food and Drug Administration (FDA), also revealed a noncausal association between COVID-19 vaccination (namely, mRNA-based vaccines) and cancer, relative to other vaccines [23]. Specifically, Seneff et al. [23] focused on two distinct approaches. One was to gather the counts for any terms that contained keywords linked to cancer, namely, “cancer,” “lymphoma,” “leukemia,” “metastasis,” “carcinoma,” and “neoplasm.” Overall, the researchers found 1,474 entries linking these terms to COVID-19 vaccines, representing 96% of all the entries for any of these terms for any vaccine in that year. The complementary approach was to find terms involving cancer in specific organs, namely, breasts, prostate, bladder, colon, brain, lungs, pancreas, and ovaries. Although all the numbers were small, the authors tabulated 534 cases of cancer of specific organs linked to COVID-19 vaccines, representing 97.3% of all the cases for any vaccine in 2021 [23].

While malignancies are generally understood to take months or, more commonly, years to progress such that the existence of a potential long-term health threat cannot be fully ascertained at present, some rapidly progressing cancers and the reawakening of dormant cancer cells (DCCs), which is associated with cancer recurrence and metastasis, are often aggressive processes that can be rapidly detected [24,25]. Identifying, understanding, and eventually preventing these potential mid- and long-term health threats is of paramount importance. To our knowledge, prospective pharmacovigilance and/or monitoring of vaccinated recipients versus matched unvaccinated controls have not been pursued in well-designed clinical trials. In addition, national estimates of cancer recurrence are not routinely collected by cancer registries although recurrence is not uncommon [26]. For some cancers, such as prostate, there are formulas that can help estimate the risk of relapse based on stage and other clinical information [26]. Yet, because recurrence rates vary depending on tumor characteristics, stage of disease, and treatment, it is not possible to predict (without population-representative data) if the cancer is completely eradicated except in hindsight. It is thus critical for all cancer survivors to be on some type of surveillance program to make sure that, if the cancer returns, this can be promptly detected. Of equal relevance is the use of animal models in understanding diseases and evaluating the safety of drugs, vaccines, food additives, and many other substances. Based on our information, no animal models exist to help understand the impact of COVID-19 vaccination on the clinical outcomes of cancer patients. Advancing research on these topics will help health authorities properly assess the risk/benefit ratio of COVID-19 vaccination in a population at increased risk of severe COVID-19 outcomes [27] as well as to draw more robust conclusions with regard to vaccination (or appropriate alternatives) in people diagnosed with cancer or with a history of prior cancer.

In sum, this article aims to highlight the pressing need to study and compare the incidence of cancer complications after COVID-19 vaccination with the incidence of similar events after SARS-CoV-2 infection (in the unvaccinated population). Focus is placed on vaccines that promote the endogenous production of SARS-CoV-2 spike (S) glycoprotein, namely, mRNA vaccines (Pfizer/BioNTech and Moderna) and adenovirus-vectorized vaccines (Johnson & Johnson and Oxford/AstraZeneca) [28]. These products have been, without doubt, the most widely used worldwide, even when more conventional alternatives (i.e., vaccines based on recombinant proteins and live and attenuated viruses) were developed. We acknowledge that other clinical and social factors resulting from the pandemic, such as adverse effects related to SARS-CoV-2 infection [29,30]; steep declines in cancer screening, diagnosis, and treatment [31]; adoption of unhealthy behaviors (i.e., increased alcohol consumption and reduced physical activity) during long pandemic lockdowns [32]; stress induced by the COVID-19 crisis [33]; and the assumption that millions of adults will remain unemployed and without health insurance, will independently contribute to cancer mortality in the months and years to come.

An earlier version of this article was previously posted to the Authorea preprint server on October 17, 2023.

## Review

**Figure 1:**
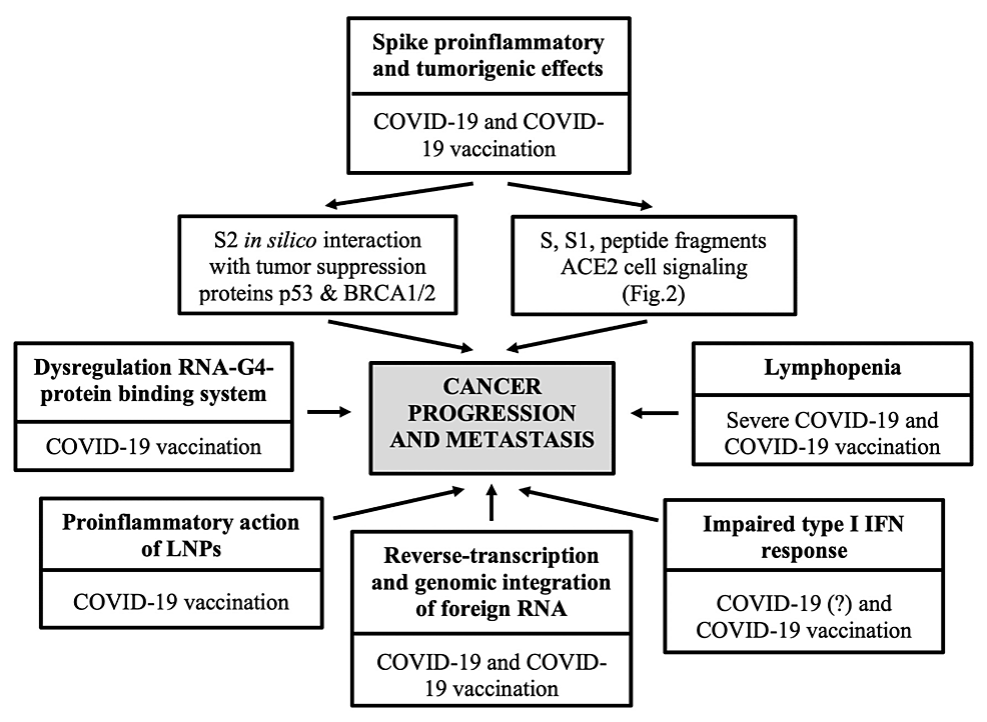
Cancer-promoting molecular mechanisms and pathways potentially mediated by SARS-CoV-2 and/or certain COVID-19 vaccines SARS-CoV-2: severe acute respiratory syndrome coronavirus 2, COVID-19: coronavirus 2019, BRCA1/2: breast cancer 1/2, ACE2: angiotensin-converting enzyme 2, LNPs: lipid nanoparticles, RNA: ribonucleic acid, IFN: interferon

SARS-CoV-2 spike glycoprotein-based vaccines, particularly mRNA vaccines, have the potential to initiate a set of biological mechanisms that may collectively generate a (transient) pro-tumorigenic environment favorable to cancer progression and/or reactivation of DCCs. These adverse effects may be attributed to the pro-inflammatory action of the lipid nanoparticles (LNPs), the impaired type I interferon (IFN) response, the translational dysregulation of cellular microRNAs triggered by structurally modified mRNA (mRNA vaccines), and/or the unique nature, expression pattern, binding profile, and pro-inflammatory and tumorigenic effects of the produced antigens, namely, the SARS-CoV-2 spike protein and/or its subunits S1 and S2 (mRNA and adenovirus-vectorized vaccines) (Figure 1).

In addition, high levels of soluble spike and/or its subunits and peptide fragments have been found in the circulation of vaccinees, where they persist for weeks or even months. It is thus plausible that the sustained and systemic distribution of spike within the human body (viral spike will not, in most cases, impact tissues and organs other than the respiratory tract) may promote a range of unforeseen interactions with angiotensin-converting enzyme 2 (ACE2), the entry receptor for SARS-CoV-2, either in its soluble circulating form or expressed in cells from various tissues and organs.

For the foregoing reasons, it is imperative to understand the effects of COVID-19 and COVID-19 vaccination on cancer cells and their microenvironment.

Lymphopenia is a hallmark of both severe COVID-19 and COVID-19 vaccination

Lymphopenia, a condition defined by abnormally low counts of lymphocytes, is a feature of severe COVID-19 compared with non-severe disease [34-36]. The possible underlying causes for the observed lymphopenia, especially the decrease in T cell counts, include T cell redistribution into infected organs, activation-induced exhaustion, apoptosis, and pyropoptosis [37]. While T cell exhaustion is observed in other viral infections [38], it seems to be more rapid, profound, and long-lasting in the setting of COVID-19. A recent study suggests that lymphopenia in severe COVID-19 patients is likely to result from SARS-CoV-2 infection of T cells in a spike-ACE2-independent manner [39]. Additionally, it has been reported that the expression of spike alone is sufficient to induce a rapid membrane fusion to produce syncytium, a type of large cells with multiple nuclei that are negative in intercellular junction molecules such as E-cadherin. Syncytia tend to internalize lymphocytes, conceivably contributing to lymphocyte loss in patients with COVID-19 [40].

Lymphopenia has also been associated with COVID-19 vaccination. Phase I/II clinical trials with the BNT162b1 (Pfizer/BioNTech) [41] and ChAdOx1 (Oxford/AstraZeneca) [42] vaccines described a dose-dependent decrease in plasma lymphocytes 6-8 days post-vaccination in 45%-46% of participants. Consistently, two preprints based on the immunization programs in Israel (BNT162b1 vaccine) [43] and England (BNT162b1 and ChAdOx1 vaccines) [44] reported an initial surge in infection risk up to nine days following vaccination. Nonetheless, T-lymphocytes specific to SARS-CoV-2 viral antigens have been shown to ultimately increase after immunization with both genetic vaccines (i.e., spike-specific T cells) and traditional platforms such as the multiantigen modified vaccinia virus Ankara (MVA)-based COVID-19 vaccine COH04S1 (i.e., membrane-, nucleoprotein-, and spike-specific T cells) [45,46]. 

Even though the molecular mechanisms that underlie lymphopenia in both COVID-19 infection and vaccination are not fully understood, lymphopenia has long been associated with increased cancer incidence and risk of malignancy [47]. Lymphocyte alterations are frequent in patients with cancer and strongly impact prognosis and survival [47,48]. Severe CD4+ T cell lymphopenia is one of the hallmarks of human immunodeficiency virus (HIV) infection. People who have HIV/AIDS are at higher risk of developing certain types of tumors (i.e., Kaposi sarcoma) than people without the disease [49-51]. CD8+ T cells have a crucial function in immune-mediated dormancy, and their depletion releases the brakes on DCCs, leading to metastatic outgrowth [52,53]. Anesthetic-induced immunosuppression can promote cancer relapses depending on the dose, duration, and timing of use [54]. Exposure to immunosuppressive drugs that prevent organ rejection in organ transplant recipients impairs cancer surveillance and facilitates the action of oncogenic viruses, increasing the post-transplant risk of neoplastic complications [55]. Analogously, organ transplant recipients accepting an organ from a cancer survivor donor might develop malignancy because exposure to the immunosuppressant drugs allows hidden latent metastases (transplanted with the organ) to spring to life [56]. Of note, 25% of cancers that developed in patients with organ transplants experience a clinical remission when the administered dose of the immunosuppressive drug is drastically reduced [57]. Remarkably, some types of cancer treatment, such as chemotherapy, radiation, and the combination of chemotherapy and immunotherapy, can also cause severe lymphopenia, which is correlated with reduced survival [47,58,59].

Given that lymphopenia, together with inflammation-related factors (described below), contributes to the creation of a microenvironment favorable to cancer progression and/or reawakening of DCCs, extreme caution is needed when recommending COVID-19 vaccination (up to five doses) [8] to oncologic patients, especially those undergoing anticancer treatment. Comprehensive studies concerning the molecular mechanisms that lead to overall lymphocyte reduction in both COVID-19 patients and vaccinees should help identify improved vaccination strategies and/or alternative interventions that prevent this major immunological abnormality and its consequences.

The SARS-CoV-2 spike glycoprotein and its S1 subunit elicit cell signaling in vitro that might be conducive to tumorigenesis in vivo

**Figure 2:**
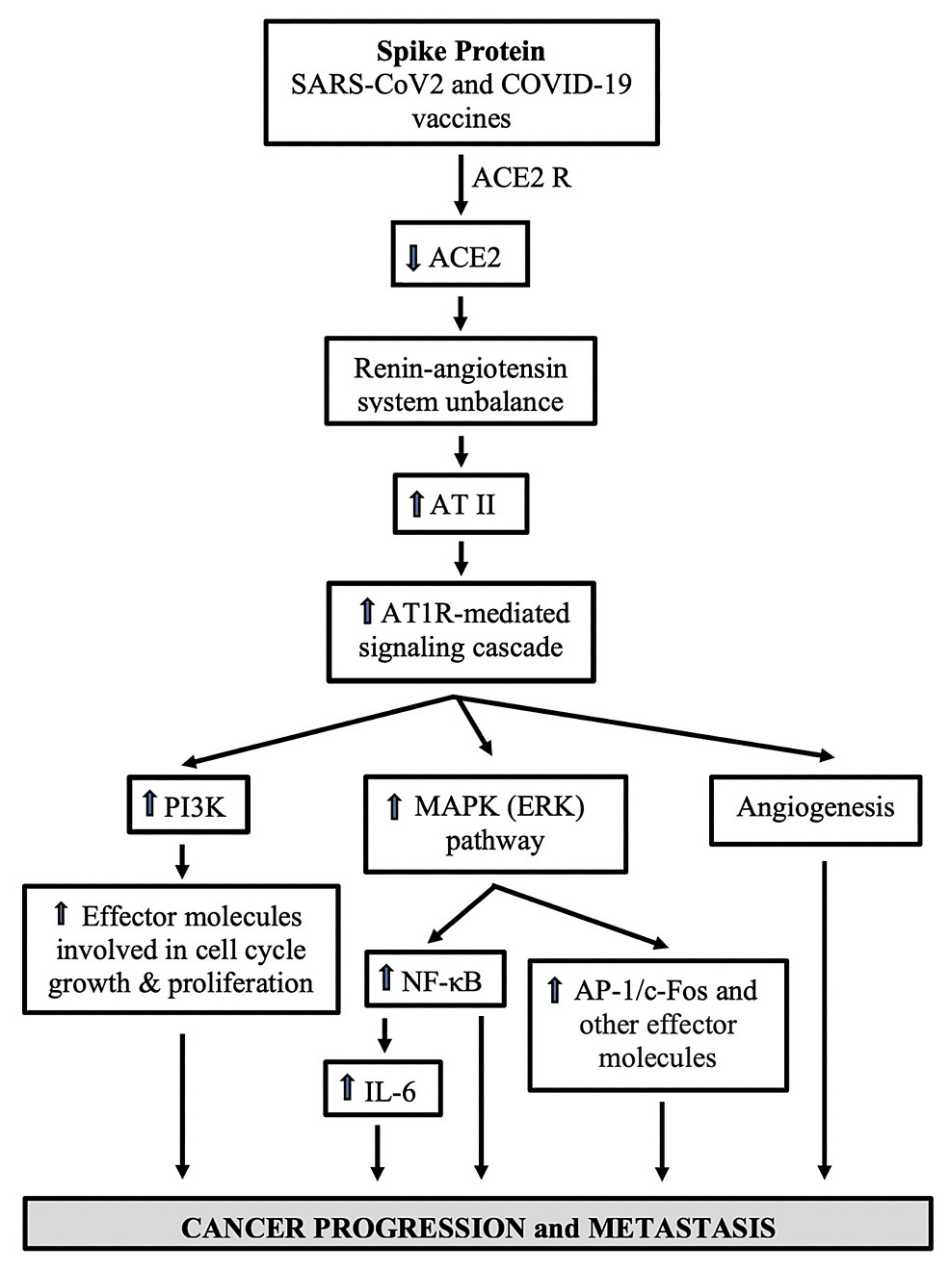
Spike-mediated ACE2 downregulation and cell signaling might promote cancer progression in COVID-19 patients and vaccinees ACE2 downregulation and its subsequent AT1R-mediated response have the potential to encourage cancer progression and metastasis through its growth-promoting and proangiogenic activities. SARS-CoV-2: severe acute respiratory syndrome coronavirus 2, COVID-19: coronavirus 2019, ACE2 R: angiotensin-converting enzyme 2 acting as an entry receptor for SARS-CoV-2, ACE2: angiotensin-converting enzyme 2, AT II: angiotensin II, AT1R: angiotensin II type 1 receptor, PI3K: phosphatidylinositol 3-kinase, MAPK: mitogen-activated protein kinase, ERK: extracellular signal-regulated kinase, NF-κB: nuclear factor-κB, IL-6: interleukin 6, AP-1: activating protein 1

SARS-CoV-2 contains a spike protein that consists of two subunits: S1 and S2. S1 aids the virus in infecting human cells by binding to ACE2, a multifunctional protein mostly expressed on the surface of many cells [60,61]. S2 mediates the membrane fusion process [62]. In addition to facilitating the entry of SARS-CoV-2 into the host cells, the interaction between spike and AEC2 elicits cell signaling in those cells expressing ACE2 [63]. Data show that, in lung vascular cells and cells implicated in the development of pulmonary arterial hypertension, the S1 subunit of spike alone activated MEK, the modulator of extracellular signal-regulated kinase (ERK) [63], which is a signal transduction mechanism for cell growth [64]. In addition, Patra et al. [65] conveyed that the full-length spike, through the downregulation of ACE2 expression, promoted an angiotensin II type I receptor (AT1R)-mediated signaling cascade, induced the transcriptional regulatory molecules nuclear factor-κB (NF-κB) and activator protein 1 (AP-1)/c-Fos via mitogen-activated protein kinase (MAPK) activation, and increased interleukin 6 (IL-6) levels in epithelial cells (Figure 2) [65].

NF-κB activation in cancer cells promotes proliferation, chemoresistance, and invasion, whereas in the tumor microenvironment, it stimulates angiogenesis and immune suppression, collectively supporting the metastatic process [66]. The Ras/Raf/MEK/ERK (MAPK) signaling cascade is frequently involved in malignancy [67]. Indeed, over 30% of all human cancers are driven by *Ras* genes [68-75]. Elevated levels of IL-6 correlate with increased rates of tumor relapse in breast cancer and head and neck cancer [76,77]. By contrast, the inhibition of IL-6/signal transducer and activator of transcription 3 (STAT3) signaling reduced cancer recurrence in preclinical models of breast, head and neck, and hepatocellular carcinoma [78-80]. The AT1R-mediated signaling cascade also activates phosphatidylinositol 3-kinase (PI3K), a component of one of the most important intracellular pathways (PI3K/AKT/mTOR) and a master regulator for cancer [67,81]. Over-activation of this pathway is present in many human malignancies and has been implicated in cancer progression. Consistently, the use of PIK3 inhibitors is a common approach in the treatment of tumors [82].

Considering that human cells sensitively respond to spike and/or its S1 subunit to elicit ACE2 cell signaling and ACE2 exerts multiple anti-tumoral and anti-invasive effects, including the inhibition of cancer angiogenesis and metastasis, the prolonged (or even transient) spike-mediated ACE2 downregulation (or loss) could per se promote tumor progression [83-86]. Remarkably, free-floating spike, S subunits, and S peptide fragments have been found to enter the circulation and persist in the body for weeks [87,88] and even months [89] following COVID-19 vaccination at concentrations comparable to those found in severe COVID-19 patients (Table 1) [89,90].

**Table 1:** Concentration and persistence in the body of spike antigens after mRNA-mediated vaccination *: [87], **: [88], ***: [89] BNT162b: Pfizer/BioNTech vaccine, mRNA-1273: Moderna vaccine

Antigen	Vaccination	Concentration (pg/mL)	Time in the body (days)
S^*^	BNT162b mRNA-1273	Days 1-2 after the first dose - median S levels: 47 pg/mL (plasma)	Present at least 1-2 days (plasma) and 60 days (germinal centers and lymph nodes) post-second dose
		Day 7 after the first dose - median S levels: 1.7 pg/mL (plasma)	
		Days 1-2 after the second dose - median S levels: 1.2 pg/mL (plasma)	
S, S1^**^	mRNA-1273	Mean S peak levels: 62±13 pg/mL (plasma)	S present up to 15 days post-first dose, undetectable after the second dose (plasma)
		Mean S1 peak levels: 68±21 pg/mL (plasma)	S1 present up to 14 days post-first dose, undetectable after the second dose, peak levels at 5 days (plasma)
S_fragments_^***^	BNT162b mRNA-1273	-	69-187 days post-vaccination (plasma)

It is hence imperative to monitor the mid- and long-term consequences of COVID-19 vaccines that introduce spike into the human body. Most importantly, appropriate experimental animal models should be developed to understand the contribution and functional implications of these signaling cascades in relation to cancer progression, recurrence, and/or sensitivity to cancer therapies.

The mRNA vaccines are designed to deactivate the host’s innate immunity via Toll-like receptors (TLRs), compromising type I IFN responses

DNA and RNA stimulate the mammalian innate immune system through the activation of TLRs, a class of proteins mostly expressed in sentinel cells (i.e., dendritic cells and macrophages) that constitute the first line of defense against invading pathogens and endogenous molecules released from dying or damaged cells [91]. TLRs trigger multiple signaling pathways involving NF-κB, IFN regulatory factors (IRFs), and MAPKs for the production of various cytokines that play important roles in many diseases, including cancer. RNA particularly signals through human endosomal TLR3, TLR7, and TLR8; however, the incorporation of modified nucleosides into the RNA molecule ablates TLR activity [92,93]. COVID-19 mRNA vaccines have all uridines in the SARS-CoV-2 spike mRNA sequence synthetically replaced by N1-methyl-pseudouridine (m1Ψ) [94,95]. Such replacement increases biological stability, promotes mRNA translation, and dramatically inhibits innate immune sensing since uncontrolled immune activation might lead to undesirable allergic reactions and anaphylactic shock [94,96].

In spite of the critical contribution of pseudouridines to mRNA COVID-19 vaccines, little is known about the biological consequences of delivering highly stabilized m1Ψ-modified mRNA within the cytoplasm of human cells. For instance, studies show that vaccination with BNT162b2 (Pfizer/BioNTech’s first candidate to receive FDA Emergency Use Authorization) significantly decreased IFN-α (type I IFN) and IFN-γ (type II IFN) production following stimulation with the TLR7/8 agonist R848 and the TL3 agonist poly I:C [97]. According to Föhse et al. [97], the decrease in the sensitivity of endosomal TLRs that interact with the transfected modified mRNA might subsequently ablate TLR3/7/8 activity and decrease cytokine production. Importantly, an effective immune response involves the induction of a robust TLR-mediated type I IFN signaling cascade as part of the innate immune system. If this response is ablated, immunopathology during lytic and latent viral infections may result [98,99]. Defects in TLR expression have been reported in people with herpesvirus infections [100,101]. Mutations in TLR3 and its downstream signaling molecules have been associated with cases of herpes simplex virus encephalitis [102,103], varicella zoster virus meningoencephalitis [102], and recurrent herpes zoster ophthalmicus [103]. Of note, an increasingly high number of herpes zoster cases has been reported following mRNA (BNT162b2 and mRNA-1273), but not adenovirus-vectorized or inactivated COVID-19 vaccination [104-109]. Such observation is consistent with an impaired TLR-mediated type I IFN response triggered by m1Ψ-modified mRNA.

Multimodal single-cell profiling of peripheral blood of patients with acute COVID-19 and healthy volunteers before and after receiving the BNT162b2 injection also revealed dramatic differences in response to both immune challenges. In COVID-19 patients, immune responses were characterized by a highly augmented type I IFN response, which was largely absent in vaccine recipients. Increased IFN signaling likely contributed to the drastic upregulation of cytotoxic genes in the peripheral T cells and innate-like lymphocytes observed in COVID-19 patients. Analysis of B and T cell repertoires disclosed that while the majority of clonal lymphocytes in COVID-19 patients were effector cells, in vaccine recipients, clonal expansion was primarily restricted to circulating memory cells [110]. Despite this, there is no ample consensus on whether type I IFN activity is robust [23,110,111] or compromised [112,113] during SARS-CoV-2 infection. For instance, contrary evidence shows that the SARS-CoV-2 S1 subunit directly suppressed the expression of ACE2 and type I IFNs in primary cells from macaque lung bronchoalveolar lavage [113], therefore contributing to SARS-CoV-2-associated lung disease. Additionally, COVID-19 diagnosis in ≥50-year-olds has been associated with an increased risk of developing herpes zoster [114,115]. This apparent controversy might be partially explained by the fine-tuning between acute antiviral immune responses that quickly achieve infection clearance through high IFN secretion and those that lead to longer and more robust inflammatory patterns (i.e., severe forms of COVID-19) with functional exhaustion of IFN responses [116]. Notwithstanding, peripheral lymphopenia (described in both severe COVID-19 patients and COVID-19 vaccinees) could alternatively (or additionally) justify the reactivation of latent herpes zoster infections in both COVID-19 patients and people who received the COVID-19 mRNA vaccines.

Remarkably, TLRs are expressed not only in immune cells but also in tumor cells, where they can both inhibit and promote malignancy [117]. Copious studies in humans and mice underline the importance of endogenous type I IFN, produced by both immune and tumor cells, in the control of tumor growth and in the response to anti-tumor therapies [118-120]. Seneff et al. [23] extensively discuss the complexity and the role of type I IFNs, particularly IFN-α, in cancer surveillance and cancer suppression. The authors point out the dazzling range of anticancer effects initiated by IFN-α through both direct (i.e., cell cycle arrest, apoptosis, and activation of natural killer and CD8+ T cells) and indirect (i.e., gene transcription activation of the Janus kinase/signal transducer and activator of transcription (JAK/STAT) pathway) mechanisms [23]. The JAK/STAT pathway is dysregulated in several hematologic malignancies, and this has been shown to increase the metastatic potential in animal models of melanoma, colorectal cancer, and lymphoma [121]. Defects in lymphocyte IFN signaling arise in patients with breast cancer, melanoma, and gastrointestinal cancer, and these defects may represent a common cancer-associated mechanism of immune dysfunction [120]. Consistently, the exogenous administration of type I IFN and/or the use of type I IFN inducers boost the innate and adaptive immune responses against solid tumors [122,123].

Impairment of type I IFN responses is also observed in other diseases, including chronic infections (i.e., HIV/AIDS) and autoimmune conditions (i.e., multiple sclerosis (MS)). By interfering with type I IFN responses, HIV-1 can circumvent host antiviral signaling and establish persistent viral reservoirs. HIV-1-mediated defects in the IFN pathway include the impairment of protein receptors involved in pathogen detection, downstream signaling cascades required for type I IFN upregulation, and expression or function of key type I IFN-inducible antiviral proteins [124,125]. Remarkably, people infected with HIV have a substantially higher risk of some types of cancer compared with the general population, including Kaposi sarcoma, non-Hodgkin lymphoma, cervical cancer [50], and, to a lesser extent, cancers of the anus, liver, oral cavity/pharynx, and lung and Hodgkin lymphoma [51]. Similarly, patients with MS who have a suppressed type I IFN signaling and respond well to IFN therapy [126,127] are also at greater risk of developing cancer than the general population [128]. This increased risk is particularly apparent for prostate, breast, colorectal, and anal cancers, as well as cancers of the trachea, bronchus, and lung.

Overall, the exceedingly complicated and pleiotropic roles of TLR and type I IFN responses in tumor biology prompt caution when introducing synthetic (i.e., m1Ψs) mRNAs for in vivo therapeutic applications. Most importantly, disrupted TLR-mediated type I IFN responses following SARS-CoV-2 infection and mRNA vaccination may not be comparable for the following reasons. First, synthetic m1Ψ-modified mRNA, unlike viral RNA, has the ability to ablate TLR activity. Second, recent studies suggest that endogenous production of synthetic spike persists for a long time (>6 months) within the human body [87-89]. Third, whereas most of the viral spike protein likely remains in the respiratory tract, vaccine-induced spike production takes place in internal organs and tissues, thus being in the position to exert more systemic effects [129]. Indeed, biodistribution studies of the BNT162b2 vaccine in animal models revealed that the vaccine does not remain at the site of injection but rather accumulates in different organs (i.e., liver, spleen, lungs, ovaries, etc.) 48 hours post-inoculation [130-133]. In this context, Bansal et al. [134] demonstrated an important role of circulating exosomes expressing spike glycoprotein on the surface for effective immunization following mRNA-based vaccination. These exosomes were detectable on day 14 following the first injection, increased following the booster dose, and considerably decreased after four months. It is thus plausible that exosomes contribute to dispersed spike (and its mRNA sequence) throughout the body, traveling via the lymph system, the vascular system, and even along nerve fibers. Finally, compliance with multiple-dose vaccine schedules at relatively short intervals [8] may conceivably increase the risk of adverse effects in vaccine recipients. Further studies should shed light on relevant TLR-dependent pro- and anti-tumorigenic pathways that may be dysregulated as a result of mRNA vaccination and/or SARS-CoV-2 infection.

Codon optimization of COVID-19 vaccines may lead to the dysregulation of the RNA-G quadruplex (G4)-protein binding system, altering the translational regulation of cellular microRNAs

The design of COVID-19 vaccines involves different types of optimizations, including codon optimization [135]. Codon optimization is a gene engineering approach that uses synonymous codon changes to increase protein production in hosts that do not naturally express the gene. This process generally increases GC content, which correlates with an increased level of transcription, possibly as a result of decreased transcriptional pausing [136]. Some authors advise that codon optimization compromises the safety and efficacy of biotech therapeutics [137]. McKernan et al. [138], Seneff et al. [23], and others describe that the significant enrichment of GC content in COVID-19 mRNA vaccines (as compared to the native SARS-CoV-2 spike mRNA) might lead to an increase of secondary structures such as the G4 motifs during translation. Specifically, McKernan et al. [138] present a series of in silico approaches such as RNAfold and QGRSMapper that show changes to the secondary structure in the vaccine-derived RNAs compared to the native virus. Of note is the increased number of G4 formations in the codon-optimized mRNA vaccines (i.e., 19 and nine G4 motifs in the Moderna and Pfizer/BioNTech mRNAs, respectively, versus four G4 motifs in the spike coding region of the SARS-CoV-2 virus). The abundance of G4 structures in the vaccinal mRNA likely amplifies the attachment of RNA-binding proteins and microRNAs that normally target human-expressed G4s for normal regulation of human gene expression. Moreover, the use of m1Ψ in the vaccinal mRNAs further obscures the folding predictions as m1Ψ promiscuous base pairing facilitates translation errors [135,139-141] and stabilizes G4s [142,143], thus exacerbating the impact of G4 formation with codon optimization [138].

Dysregulation of the RNA G4-protein binding system might dramatically downregulate cellular microRNA expression, which is involved in many pathological conditions such as cardiovascular disease, the onset of neurodegeneration, and cancer progression [23]. One example, vital for cellular normal housekeeping, is that of mouse double minute 2 (MDM2) homolog, which is a physical negative regulatory protein of p53 (p53 is a well-known tumor suppressor protein described below). Dysregulation of microRNAS that control the intricate interplay between MDM2 and p53 predictably leads to an increased risk of a range of cancers [23,138,144-146]. Another example is the amplification of RNA G4 repeats in amyotrophic lateral sclerosis/frontotemporal dementia (ALS/FTD) (*C90RF72* gene) and Fragile X syndrome (*FMR1* gene) [147]. In these diseases, changes in the expression levels of or mutations in RNA G4-binding proteins are also reported, suggesting that these proteins cannot exert their critical function for normal neuron physiology when mutated or in cells with RNA G4 expansions [147].

Largely, these observations highlight the evolved complexity of codon usage and challenge the scientific bases for codon optimization in human therapeutics.

The LNPs used in mRNA vaccines are highly inflammatory in mice

LNPs are a vital component of mRNA-based COVID-19 vaccines, playing a key role in improving the in vivo stability of mRNA and enhancing delivery to the cytosol of antigen-presenting cells [148]. LNPs consist of four main components: a neutral phospholipid, cholesterol, a polyethylene-glycol lipid, and an ionizable cationic lipid [149].

The highly inflammatory properties of cationic LNPs have been known since 2010 [150]. A recent report [150] specifically showed that the LNPs used in preclinical nucleoside-modified mRNA COVID-19 vaccine studies are highly inflammatory in mice. Intradermal injection of these LNPs led to massive infiltration of neutrophils, rapid and robust activation of diverse inflammatory pathways, and production of various inflammatory cytokines and chemokines. Intranasal delivery led to similar inflammatory responses in the lung [151]. While the intrinsic adjuvant activity of LNPs may contribute to eliciting protective immunity, the uncontrolled activation of various distinct and convergent inflammatory pathways and the secretion of inflammatory cytokines and chemokines might lead to severe inflammation and cytotoxicity. Extensive studies are therefore needed to map the interactions between cationic LNPs and intracellular pattern recognition receptors to unravel integrated and multifaceted mechanisms by which these lipids induce inflammasome activation [152]. In addition, while it is probable that intramuscular injection of the COVID-19 vaccine LNP-mRNA complexes triggers similar responses in humans [151], the exact nature of such responses and how much they overlap with the inflammatory signatures documented in mice remain unknown. Relevantly, adenovirus-vectorized injections, unlike mRNA vaccines, do not induce severe innate immune responses (i.e., cytokine storm), hyperinflammation, or major damage in the targeted cells [153]. Conversely, severe COVID-19 (which affects about 5% of the SARS-CoV-2-infected population) [154] triggers a cytokine storm in pulmonary tissues that may be accompanied by immunopathology, viremia, and systemic multiorgan collapse [155-157].

In the context of cancer, inflammation predisposes the development of disease and promotes all stages of tumorigenesis [158]. Tumor-extrinsic inflammation is caused by many factors including bacterial and viral infections, autoimmune diseases, obesity, tobacco smoking, asbestos exposure, and excessive alcohol consumption [158]. Around 15%-20% of all cancer cases are preceded by infection, chronic inflammation, or autoimmunity at the same tissue or organ site [158-164]. In such cases, cancer-promoting inflammation is induced and exists long before tumor formation. In contrast, cancer-intrinsic or cancer-elicited inflammation can be triggered by cancer-initiating mutations, contributing to malignant progression through the recruitment and activation of inflammatory cells [158]. Both extrinsic and intrinsic inflammation can result in immunosuppression, thereby providing a preferred background for tumor development. Of note, neutrophils are actively involved in a network of inflammatory reactions that promote all the stages of tumor initiation, progression, angiogenesis, and metastasis [165-170]. Neutrophils form neutrophil extracellular traps (NETs) that, when dysregulated, lead to the exacerbation of inflammation [171,172], unconstrained cancer progression, reawakening of DCCs, and metastatic dissemination, both in animal models and cancer patients [173]. In addition, the tumor microenvironment, which is largely orchestrated by inflammatory cells, fosters the proliferation, survival, and migration of neoplastic cells. Markedly, inflammatory responses are aggravated on a background of pre-existing inflammatory conditions, as was recently demonstrated in a mouse model after the administration of mRNA-LNPs [174]. This effect was proven to be specific to the LNPs, acting independently of the mRNA cargo. Given that LNPs often accumulate in tumors, due to enhanced permeability and retention effect (EPR) [175-178], protecting cancer cells from transformation-related stress stimuli, including inflammation and the pro-tumorigenic action of NETs, is of paramount importance. Understanding the interactions between LNPs and neutrophils [179] should thus be critical for the development of safe and effective nanomaterials.

Potential reverse-transcription and genomic integration of foreign RNA are a source of genomic instability

A new study by Acevedo-Whitehouse and Bruno [180] discusses the possibility that parts of the SARS-CoV-2 genome might undergo reverse-transcription and genomic integration within infected cells, leading to persistent transcription of the integrated sequences. This hypothesis is based on an in vitro study that detected the presence of reverse-transcribed copies of SARS-CoV-2 sequences in transfected human cells and found active transcription of the integrated sub-genomic segments [181]. Acevedo-Whitehouse and Bruno speculate that the same phenomenon could occur in human cells that received COVID-19 mRNA vaccines. Indeed, a recent study by Aldén et al. [182] reported that an endogenous retrotransposon, namely, long interspersed nuclear element-1 (LINE-1), was unsilenced following BNT162b2 mRNA entry to the cell. This led to the reverse-transcription of full-length vaccine mRNA sequences and subsequent nuclear entry.

If these results are confirmed in vivo, the sustained activity of unsilenced LINE-1, which is normally repressed in somatic cells, might increase the risk of insertional mutagenesis of the reverse-transcribed molecules, which, in turn, might disrupt coding regions, enhance the risk of mutations in tumor suppressor genes, and lead to sustained DNA damage in cells and tissues targeted by the vaccine [180]. LINE-1 retrotransposition is indeed a major hallmark of cancer [183] and correlates with p53 mutations, copy number alterations, and cell cycle S phase checkpoints [184]. Importantly, the activation of LINE-1 increases the risk of epithelial-mesenchymal transition and metastasis in epithelial cancer, which accounts for 80%-90% of all known human cancers [185]. There is hence a pressing need for clarity on the potential COVID-19- and COVID-19 vaccine-induced activation of LINE-1 and its repercussions in cancerous and/or precancerous cells with intrinsic high levels of LINE-1 expression.

Moreover, if SARS-CoV-2 spike mRNA vaccine sequences are reverse-transcribed, integrated into the genome of targeted cells, and expressed as chimeric transcripts that combine viral and cellular sequences, dysregulation of the RNA G4-protein binding system might further promote malignancy. Indeed, experimental studies and bioinformatics predictions support the view that G4s are involved in different cellular functions associated with both DNA processes (i.e., telomere elongation, recombination, and transcription) and RNA posttranscriptional mechanisms (i.e., pre-mRNA processing, mRNA turnover, targeting, and translation) [186]. As previously noted, an increasing number of different diseases (i.e., neoplastic transformation and neurodegeneration) have been associated with the inappropriate regulation of RNA G4s, exemplifying the potential importance of these structures in human health. Notably, G4 structure formation, if not regulated efficiently, can stimulate genome instability, inducing mutations, deletions, and complex gross chromosomal rearrangements [187]. A computational study that compared the location of potential G4-forming sites with cancer-associated breakpoints revealed a significant overlap, particularly in those cancers that harbor mutations in *TP53* (the gene that codes for p53). This is underlined by computational studies in melanoma cells that linked G4 regions with mutational hot spots [188]. Additionally, Hänsel-Hertsch et al. [189] identified a direct correlation of G4s with mutational changes in different breast cancer entities. This supports the notion that G4 formation stimulates and influences mutation rates in different cancers.

The S2 subunit of SARS-CoV-2 spike glycoprotein interacts with tumor suppressor proteins p53 and breast cancer 1/2 (BRCA1/2) in silico

Using bioinformatics (in silico) analyses, Singh and Bharara [190] proved that the S2 subunit of SARS-CoV-2 strongly interacts with well-known tumor suppressor proteins p53 and BRCA1/2, which are frequently mutated in human cancers. These proteins provide a major barrier to neoplastic transformation and tumor progression by their unique ability to act as extremely sensitive collectors of stress inputs and to coordinate a complex framework of diverse effector pathways and processes that protect cellular homeostasis and genome integrity. p53 and BRCA1/2 act predominantly in the cell nucleus regulating cell cycle progression, DNA damage repair and recombination, and gene transcription [191-193]. However, these proteins also play critical roles in the cytoplasm, triggering apoptosis and inhibiting autophagy, thereby contributing to their mission as tumor suppressors [194,195]. Wild-type p53 has been reported to be abnormally sequestered in the cytoplasm of a subset of primary human tumors [196]. A myriad of cancer-associated mutations that disrupt the nuclear targeting of BRCA1 restrict the protein to the cytosol and diminish its nuclear function in homologous recombination repair of DNA breaks [197]. Notably, BRCA1 cytosolic accumulation promotes breast cancer metastasis [198] and independently predicts survival, tumor grade, and recurrence in low-grade basal-like sporadic breast cancers [199].

If, as in silico, the S2 subunit of spike interacts with tumor suppressor proteins in vivo, such a demonstration would have implications not only for the long-term health of those impacted by COVID-19 but also of those who received COVID-19 vaccination and repeated booster doses. Indeed, both mRNA and adenovirus-vectorized vaccines carry the genetic material that instructs the host cells to express spike. As described above, biodistribution studies of the BNT162b2 vaccine revealed its accumulation in different organs 48 hours post-inoculation [130-134]. Most importantly, LNPs, which are a vital component of the mRNA vaccines, preferentially accumulate in tumor tissue over healthy tissue due to the EPR effect [175-178]. Based on these findings, it is essential to decipher the range, detailed role, and biological consequences of the potential interactions between S2 and tumor suppressor proteins (i.e., p53 and BRCA1/2) in COVID-19 patients and vaccinees, particularly if these interactions confer a selective advantage (i.e., promotion of cancer cell survival, invasion, metastasis, and chemoresistance) to cancer and/or precancerous cells.

Cancers associated with TP53 mutations include breast cancer, bone and soft tissue sarcomas, brain tumors, and adrenocortical carcinomas. Other less frequent cancers include leukemia, stomach cancer, and colorectal cancer [200]. Cancers associated with impaired BRCA1 activity include breast, uterine, and ovarian cancer in females, prostate and breast cancer in males, and a modest increase in pancreatic cancer for both males and females [201,202]. The most commonly reported cancers with BRCA2 mutations include pancreas, prostate in males, and melanoma [203].

Dysregulation and/or aberrant changes in p53 levels/activity [204,205] as well as cytoplasmatic sequestration of BRCA1 [206] have also been linked to neuronal dysfunction. Therefore, the potential in vivo interaction between S2 and tumor suppressor proteins might have consequences not only for rapidly cycling cancer cells but also for non-cycling cells (notably neurons) and thus for long-latency neurodegenerative diseases [207,208].

Cluster of differentiation 147 (CD147) transmembrane protein, a novel entry route for SARS-CoV-2 infection to host cells, is correlated with various cancers

Recently, a novel SARS-CoV-2 entry route was proposed, namely, utilization of CD147 transmembrane glycoprotein [209]. Despite lesser affinity toward the spike protein of SARS-CoV-2, as compared to ACE2, CD147 might be a complementary receptor in mediating virus infection [210]. Although unequivocal evidence supporting a direct interaction between spike and CD147 is currently missing [211], confirmation of CD147 as a novel SARS-CoV-2 viral target might have serious implications for oncologic patients. CD147 has been correlated with various cancers [212,213] and has been shown to participate in the upregulation of the tumor microenvironment and cancer progression by several mechanisms, namely, the control of glycolysis and its well-known ability to induce proteinases leading to matrix degradation, tumor cell invasion, metastasis, and angiogenesis [214]. As previously described for ACE2, the possible interaction of SARS-CoV-2 spike glycoprotein with CD147 receptors could, through the activation of tumorigenic pathways, pave the way for cancer progression and/or recurrence.

Consideration of COVID-19 vaccination in people with cancer or a history of cancer

COVID-19 vaccination is the largest emergency immunization campaign ever attempted in human history [215]. Although the pandemic has largely vanished from public discourse, approximately 2,000-3,000 Americans are still dying from COVID-19 every week [216], and the same trend is observed in the UK [217], possibly because some collectives (i.e., older people, people with comorbidities, and immunocompromised individuals) still face a high risk of serious illness, particularly now that nearly every part of society has returned to normal. Therefore, the protection of millions continues to be a tremendous challenge and responsibility. While vaccines may have had a significant impact in averting deaths, serious health outcomes from vaccines may go unrecognized in clinical trials and/or passive surveillance systems such as VAERS, especially if they are mid-/long-latency and do not require immediate hospitalization. In this context, we have shown that SARS-CoV-2 spike glycoprotein-based vaccines have the potential to interact with tumor suppressor proteins, promote inflammation, activate oncogenic pathways, and disrupt the fine-tuning of the immune response. These dysregulated mechanisms and signaling pathways underlie most types of cancer. Furthermore, the genotoxic potential of spike was recently studied in guppy (*Poecilia reticulata*, also known as millionfish or rainbow fish) adults exposed to SARS-CoV-2 spike fragments dispersed in freshwater [218]. Exposure to these peptides induced genomic instability and DNA damage in circulating erythrocytes of *P. reticulata*, which were in correlation with a redox unbalance marked by increased malondialdehyde (MDA) levels in the liver and brain, as well as by suppressing the antioxidant activity of hepatic superoxide dismutase (SOD) and catalase (CAT).

While we understand that much of the discussion about cancer and COVID-19 vaccination was done under high pressure to protect this cohort from severe disease and death, a more balanced risk/benefit evaluation is urgently needed. This is especially relevant for people with poor immune responses, such as those with hematologic malignancies [219,220], for which the benefits of vaccination are dubious and the cumulative risks of successive boosters are unknown (although conceivably increased with each dose received). Of particular concern is the observation that some anticancer drugs render COVID-19 vaccines ineffective [221,222]. In addition, the coadministration of complex anticancer regimes and COVID-19 vaccines [222-224] might pave the way for intercurrent or synergistic toxic effects. Indeed, a recent article [224] on the effects of BNT162b2 vaccine in oncologic patients under checkpoint inhibitors (CPIs) describes that CPI immunotherapy resulted in a constant and variable increase of all COVID-19 vaccination side effects, which is alarming. There is thus a concern that the simultaneous use of immunotherapy and COVID-19 vaccines boosts the body’s immune response, resulting in enhanced immune-related adverse events. Moreover, reactive axillary lymphadenopathy secondary to COVID-19 vaccines may mimic cancer metastasis, posing diagnostic dilemma and increasing anxiety in patients with breast cancer, head and neck cancers, lymphoma, and melanoma of the back and upper extremities, which are all malignancies that have a predilection for metastasizing to these lymph node stations [225-229]. Precisely, a breast clinic in Israel recently reported a 394% increase in lymphadenopathies when compared to previous years, and a study that included 169 Israeli patients undergoing a positron emission tomography-computed tomography (PET-CT) scan 7-10 weeks after receiving the second dose of the BNT162b vaccine described persistent unilateral lymphadenopathy in 29% of patients [225]. In contrast, a few rare cases of temporary or prolonged cancer remission after COVID-19 [230] and mRNA-based COVID-19 vaccination [231] have been reported, possibly as a result of the intense immune-inflammatory response that may have prompted anticancer immunity in these individuals. Overall, cancer is one of the most complex, heterogeneous, and dynamic human diseases [232,233], and as such, a universal “one-size-fits-all” approach is flawed.

Unfortunately, most current cancer statistics worldwide (i.e., Japan, Australia, Canada, and Europe) do not extend beyond 2020 [234-239] unless they are estimates or projections. This makes it imperative to build global pharmacovigilance databases that help in making decisions based on the best evidence available at each moment. In the US, from January 7, 2018, to July 2, 2022, the CDC Mortality and Morbidity Weekly Reports (MMWR) listed approximately 13,000 cancer deaths per week (range: 12,221-14,845), with peaks occurring in January 2021 (14,284 deaths) and January 2022 (14,845 deaths) [240]. While the public health agency specified that the number of cancer deaths (with cancer as the underlying cause) slightly increased from 2018 to 2022, it mostly attributed the excess cancer deaths to non-cancer underlying causes, such as COVID-19. Indisputably, the cancer mortality peaks observed in 2021 and 2022 correlate well with COVID-19’s winter surges. However, they also follow two major COVID-19 vaccination and booster campaigns. As noted earlier, both SARS-CoV-2 and SARS-CoV-2 spike protein-based vaccines promote the production of spike within human cells, which, in light of the above, might facilitate malignant transformation. Chaotic death recordings during pandemic waves might have also created a distortion of facts, misguiding efforts to prevent leading causes of cancer (and other) deaths. Of note, research has found that, even under normal circumstances, critical errors in death certificates are quite common in the US, with the frequency of errors ranging from 18% to 85% or higher in hospital-based studies [241].

In short, despite the fact that many institutions [242,243] and authors [244,245] maintain that COVID-19 vaccines are safe and (partially) effective in patients with cancer, these claims are unsupported, and recommendations are largely inferred from vaccine safety and effectiveness in the general population, performance of other vaccines in patients with cancer, and immune alterations inherent in current cancer treatments [246]. Given the converging evidence of temporal association and biological plausibility, the contribution of genetic COVID-19 vaccines to cancer progression and recurrence cannot be excluded at present. Yet, one might argue that the oncogenic potential of spike should also be exerted during SARS-CoV-2 infection. While this is partially true, we already discussed that COVID-19 genetic vaccines and, in particular, mRNA injections are radically different from SARS-CoV-2 viral infection. Hence, the role of COVID-19 vaccination and SARS-CoV-2 infection in the pathways that potentially promote malignancy may not be comparable and merit further investigation. In addition, if harm can be conclusively attributed to the LNP vehicle itself and/or to the synthetic modified mRNA (regardless of the toxicity, or lack thereof, of spike), this may have implications for the development of new mRNA products based on the same core technology [247].

In view of the current state of the art, our suggestion is that individuals with cancer or a history of cancer should receive the genetic COVID-19 vaccines only if the benefits clearly outweigh any risks and after careful evaluation case by case. Most importantly, there is the possibility that cancer risk is dose-dependent. According to the “multi-hit model of carcinogenesis” proposed by Sutherland and Bailer [248], it takes multiple different hits or insults to cells and their genetic machinery to cause a normal cell to become cancerous. Since the COVID-19 vaccines are not a primary series for protection but rather periodic (every six months) injections without any stopping point, it is possible that only those with multiple immunizations (and/or high risk for cancer or cancer relapse) would be at higher risk of malignancy. Multidisciplinary clinical and basic research comparing the cellular and molecular basis of COVID-19- and COVID-19 vaccine-induced oncogenic effects may help rebalance the risk/benefit profile of these products. Direct approaches, such as the use of animal models, should take advantage of the recent development of mice expressing human ACE2 receptors [249,250] and the availability of cancer mouse models [250]. Studies investigating the efficacy and safety of COVID-19 vaccination in cancer patients, both prospectively and retrospectively, are strongly encouraged. Patient- and treatment-associated factors merit specific consideration. The need for more reliable databases that include widely measured immune parameters as well as data on spike protein levels in blood has been pointed out by others [251]. Taken together, these studies should provide robust data to guide clinical implementation, including the development of therapeutic alternatives (i.e., LNPs with different chemistry, a closed form of spike not prone to ACE2 binding [252], non-spike targeting vaccines [253], platforms such as COH04S1 [254] with high tolerability and immunogenicity in immunosuppressed individuals, non-pharmacological interventions [255], etc.), for those who do not benefit from active COVID-19 vaccination (and those who are allergic to some of the vaccine components).

## Conclusions

This comprehensive literature review aims to highlight the potential that COVID-19 genetic vaccines, particularly mRNA vaccines, have to fulfill the multi-hit hypothesis of oncogenesis as originally proposed by Sutherland and Bailor in 1984, in that they elicit a pro-tumorigenic milieu favorable to cancer progression and/or (metastatic) recurrence. Proving this potentiality wrong is a necessary step toward satisfying the first principle of medicine: “primum non nocere” (“first, do no harm”). Indeed, all global crises pose tremendous challenges to health and welfare; yet, such exceptionalities should not be a justification for lowering scientific standards. This is particularly relevant for prophylactic drugs intended to protect vulnerable high-risk populations across the world. Precisely, the success of the novel mRNA-based vaccines against COVID-19 has created a widespread interest in mRNA technology as a solution to some of the deadliest infectious diseases (i.e., malaria, tuberculosis, and HIV/AIDS) for which an effective and easily deployable vaccine is urgently needed. However, because some of the outlined pro-oncogenic mechanisms are antigen-independent, current safety concerns should be promptly addressed before mRNA-based nanomedicines further transform the way diseases are managed and prevented in the future.
